# Effect of Cold Storage on the Viable and Total Bacterial Populations in Human Milk

**DOI:** 10.3390/nu14091875

**Published:** 2022-04-29

**Authors:** Lisa F. Stinson, Michelle L. Trevenen, Donna T. Geddes

**Affiliations:** 1 School of Molecular Sciences, The University of Western Australia, Perth, WA 6000, Australia; donna.geddes@uwa.edu.au; 2 Centre for Applied Statistics, The University of Western Australia, Perth, WA 6000, Australia; michelle.trevenen@uwa.edu.au

**Keywords:** human milk, microbiome, bacteria, storage, viability, expressed breast milk

## Abstract

Expression and cold storage of human milk is a common practice. Current guidelines for cold storage of expressed milk do not take into account the impact on the milk microbiome. Here, we investigated the impact of cold storage on viable bacterial populations in human milk. Freshly expressed milk samples (*n* = 10) were collected and analysed immediately, stored at 4 °C for four days, −20 °C for 2.25 months and 6 months, and −80 °C for 6 months. Samples were analysed using propidium monoazide (PMA; a cell viability dye) coupled with full-length 16S rRNA gene. An aliquot of each sample was additionally analysed without PMA to assess the impact of cold storage on the total DNA profile of human milk. Cold storage significantly altered the composition of both the viable microbiome and total bacterial DNA profile, with differences in the relative abundance of several OTUs observed across each storage condition. However, cold storage did not affect the richness nor diversity of the samples (PERMANOVA all *p* > 0.2). Storage of human milk under typical and recommended conditions results in alterations to the profile of viable bacteria, with potential implications for infant gut colonisation and infant health.

## 1. Introduction

Human milk contains a number of important bioactive components, including a low biomass of bacteria [1] which contribute to the seeding of the infant oral and gut microbiomes [2,3,4,5,6]. Expression and cold storage of human milk is a commonplace practice. Mothers may express milk for their infants in order to return to work outside the home, share feeding, feed their hospitalised infant, or feed multiple infants. In the U.S. 85–91% of breastfeeding mothers have expressed milk for their infant at some point [7,8]. Similar figures have been reported in Australia, where 69–98% of mothers have expressed milk for their infants [9,10]. However, the effect of typical cold storage on the human milk microbiome is not well understood. The Academy of Breastfeeding Medicine (ABM) provides guidelines for storage of expressed milk: four hours at room temperature (16–29 °C), four days in the refrigerator (4 °C), and six months in the freezer (≤−4 °C) [11]. However, these recommendations are based on bacterial growth and degradation of milk components, including immune cells [12], lipids [13], and antioxidants [14], and do not take into account the effect of cold storage on the viability of the milk microbiome. 

The stability of the human milk microbiome under typical cold storage conditions has been poorly characterised. To date, limited studies have attempted to answer this question, and have arrived at conflicting conclusions. Marín et al. analysed 34 milk samples fresh (processed immediately), and after two, four, and six weeks of storage at −20 °C [15]. They found no difference in bacterial colony counts across a range of culture media at any time point, suggesting that storage at −20 °C has no discernible effect on the milk microbiome. Ahrabi et al. collected 40 milk samples over 1–2 pumping sessions that occurred within four hours of one another [16]. Baseline samples were frozen at −80 °C, while stored samples were frozen immediately at −20 °C or stored at 4 °C for 72 h then frozen at −20 °C for one, three, six, or nine months. Both total bacterial and gram-positive bacterial colony counts approached zero after just three months of storage, with a greater decline seen in samples that were refrigerated and then frozen. However, an initial period of up to four hours of storage at room temperature during collection, and the use of a frozen sample to represent the baseline community may have influenced these results. Slutzah et al. analysed 36 milk samples fresh (processed immediately), and after storage at 4 °C for one, two, three, and four days [17]. There was no change in total nor gram-negative bacterial colony counts; however, a decline in gram-positive bacterial colony counts over the course of storage was observed. Collectively, the evidence on the effect of cold storage on bacterial communities in human milk is contradictory.

To better characterise the effect of cold storage on the human milk microbiome, a molecular approach is required. This will allow a higher resolution analysis of human milk bacterial communities under various storage conditions. However, DNA-based methods, such as 16S rRNA gene sequencing, cannot differentiate viable bacterial cells from dead cells, potentially leading to spurious results. Here, a combination of propidium monoazide (PMA; a cell viability dye [18]) and full-length 16S rRNA gene sequencing was used to analyse the effect of cold storage on both the total bacterial DNA profile, and the viable bacterial profile of human milk. To ensure relevance of the data, expressed milk samples were stored according to ABM guidelines (four days at 4 °C and six months −20 °C [11]), typical lab conditions (−80 °C for six months), and under home storage conditions (according to the results of a survey of milk storage practices).

## 2. Materials and Methods

### 2.1. Survey of Milk Storage Practices

Breastfeeding mothers of healthy infants aged 1–12 months who expressed milk for their infant were invited to fill out a survey of their milk storage practices. This survey was approved by the University of Western Australia’s Human Research Ethics Committee (RA/4/1/2369) and all participants provided informed consent. The survey consisted of four questions:

1. Do you store your expressed breast milk in the fridge?
2. If yes, what is the usual amount of time that you store your milk in the fridge?
3. Do you store your expressed breast milk in the freezer?
4. If yes, what is the usual amount of time that you store your milk in the freezer?

### 2.2. Sample Collection and Storage

Milk samples were collected from lactating women (*n* = 10; 1–12 months post-partum), as previously described [19]. These participants were not the same as those who answered the questionnaire. All participants provided informed consent and The University of Western Australia’s Human Research Ethics Committee approved the study (RA/4/1/2369). An amount of 50 mL of expressed milk was collected using a Symphony electric breast pump (Medela AG, Baar, Switzerland) and sterilized pump kits. Each sample was split into ten 1 mL aliquots and either processed immediately, or stored at 4 °C for four days, −20 °C for a representative “home storage” quantity of time (according to the results of the survey), −20 °C for six months, or −80 °C for six months. At each time point two aliquots from each mother were analysed: one with a PMA pre-treatment to assess the viable microbiome, and one without PMA to assess the total bacterial DNA profile.

### 2.3. PMA Treatment

Human milk samples were centrifuged at 10,000× *g* for 10 min at 4 °C. For the non-PMA treated aliquots, DNA was immediately extracted from the cell pellet. For the PMA-treated aliquots, samples were treated with PMA (PMAxx™, Biotium) as previously described [19], followed by DNA extraction from the cell pellet.

### 2.4. DNA Extraction

DNA was extracted as previously described [19] within a sterile laminar flow hood. Certified sterile and DNA-free plasticware was used for all steps.

### 2.5. PacBio Sequencing

The full-length 16S rRNA gene was amplified using the primers 27F (5′-AGRGTTYGATYMTGGCTCAG-3′) and 1492R (5′-RGYTACCTTGTTACGACTT-3′), as previously described [19,20]. PCR reagents were decontaminated using the ArcticZymes PCR decontamination kit prior to use. Certified sterile and DNA-free plasticware was used for all steps, and all work was carried out in a sterile laminar flow hood. Primary PCR products were barcoded as previously described [19,20]. Barcoded samples were pooled in an equimolar concentration and the pool was gel purified using the QIAquick Gel Extraction Kit.

The purified, barcoded DNA pool was sequenced at the Australian Genome Research Facility (University of Queensland, QLD, Australia) using the PacBio Sequel II system.

### 2.6. Sequence Processing

Sequence data was processed using mothur version 1.44.1 [21], as previously described [19].

### 2.7. Statstistical Analysis

#### 2.7.1. Survey Responses

Survey responses are summarised as means, standard deviations (SDs), and ranges.

#### 2.7.2. DNA Quantification

DNA quantity was skewed so analysis was performed after a log-transformation. Medians and interquartile ranges (IQR) are reported. A linear mixed model was performed with an outcome of (log-transformed) DNA quantity, fixed factors of PMA treatment, storage condition and their interaction, and a random effect of participant ID. Pairwise comparisons were back-transformed to ratios, with associated 95% confidence intervals (CIs), such that interpretation may be on the original DNA quantity scale. *p*-values of pairwise comparisons from this model, and all further models in this paper, were adjusted across all outcomes to control the false discovery rate using the Benjamini–Yekutieli correction [22]. As such, adjusted *p*-values are presented.

#### 2.7.3. Alpha Diversity

Alpha diversity was assessed using Shannon diversity and richness (number of OTUs). Means and standard deviations (SDs) are reported for the Shannon diversity, whilst medians and IQRs are reported for the richness. Linear mixed models were performed with outcomes of Shannon diversity and log-transformed richness. Fixed effects of PMA treatment, storage condition, and their respective interaction were included in the models, along with a random effect of participant ID. Adjusted *p*-values are presented.

#### 2.7.4. Beta Diversity

Differences in beta diversity were assessed by performing PERMANOVA on Bray–Curtis distances. Fixed effects of PMA treatment, storage condition, and their interaction, as well as a random effect of participant ID were included in the model. Adjusted *p*-values are provided. A principal correspondence analysis (PCoA) was performed, and the first two principal component axes are plotted.

#### 2.7.5. Relative Abundance Analysis

For the relative abundance analysis, results from OTUs which made up ≥1% of the total relative abundance in the samples and had a prevalence of >10% amongst mothers, are reported. Analysis was performed at the OTU level, and taxonomic assignments for each OTU were established using BLAST [23]. Relative abundances were analysed using generalised additive models for location, scale, and shape with a zero-inflated beta family. PMA treatment, storage condition, and their interaction were considered as fixed effects, and participant ID was included as a random effect. Adjusted *p*-values are provided.

## 3. Results

### 3.1. Assessment of Milk Storage Practices

In order to ascertain the conditions under which expressed human milk was stored in the home, breastfeeding mothers of healthy full-term infants were invited to answer a survey assessing the length of time that expressed milk was stored in the home refrigerator or freezer. The survey received 43 responses. Two responses were discarded due to qualitative answers (e.g., “months” or “days”), leaving 41 for analysis. Of the 41 respondents, two stored their milk in the fridge only, and three stored their milk in the freezer only. On average, participants stored their milk in the fridge for 1.8 days (SD 1.2 days, range 5 h–6 days) and in the freezer for 2.25 months (SD 1.74 months, range 4 days–9 months) (Table 1). Based on these results, 2.25 months at −20 °C was chosen as the home storage condition.

### 3.2. Cold Storage Reduces the Yield of DNA from Viable Cells

**Figure 1:**
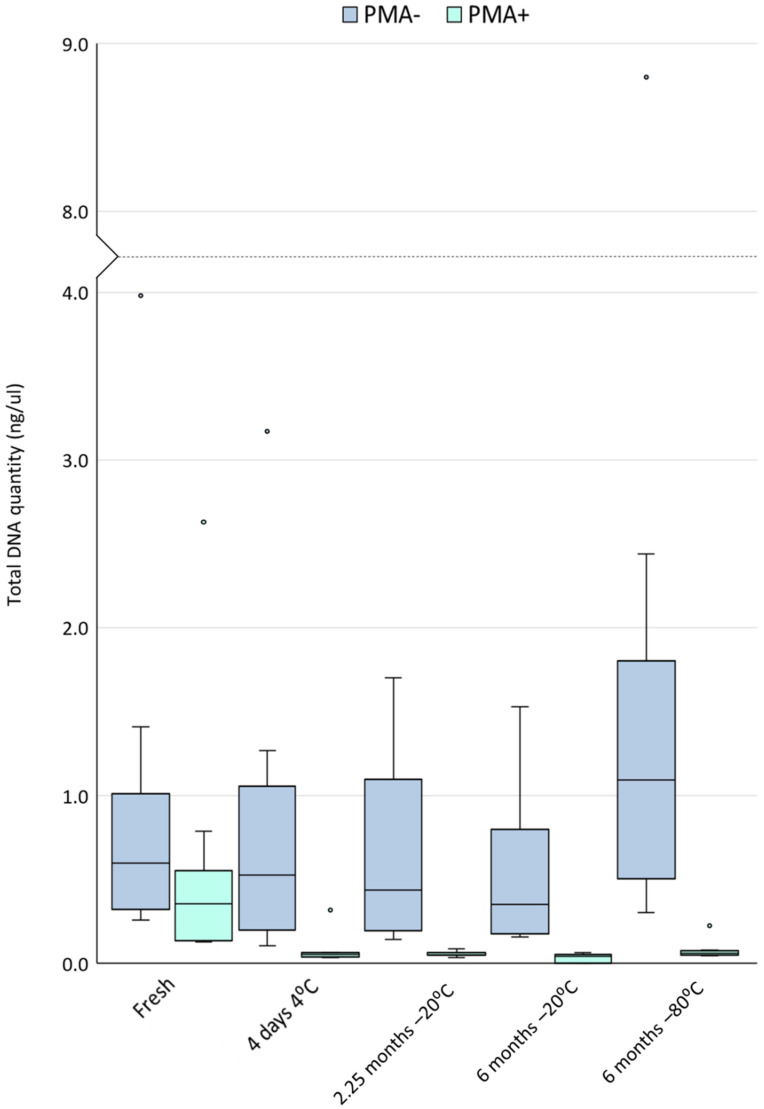
DNA concentration (ng/µL) of human milk samples under different storage conditions (*n* = 10). Dark blue bars represent total DNA from viable and non-viable cells (non-PMA treated samples). Light blue bars represent DNA from viable cells only (PMA-treated samples). Boxes are interquartile range, whiskers are range, and inner lines are medians.

The median yield of DNA from the fresh milk samples analysed here was 0.60 ng/µL (IQR 0.55, min: 0.26, max: 3.98). However, a significant portion of this DNA originated from non-viable cells. The total quantity of DNA originating from viable cells (PMA-treated aliquots) was 0.36 ng/µL (IQR 0.34, min: 0.13, max: 2.63). A detailed comparison of fresh PMA-treated and untreated samples is the subject of another publication [19]. Regardless of storage condition, PMA-treated samples yielded significantly less total DNA than untreated samples, suggesting that a significant quantity of DNA in these samples originated from non-viable cells (fresh *p* = 0.008, all other storage conditions *p* < 0.0001) (Figure 1).

**Figure 2:**
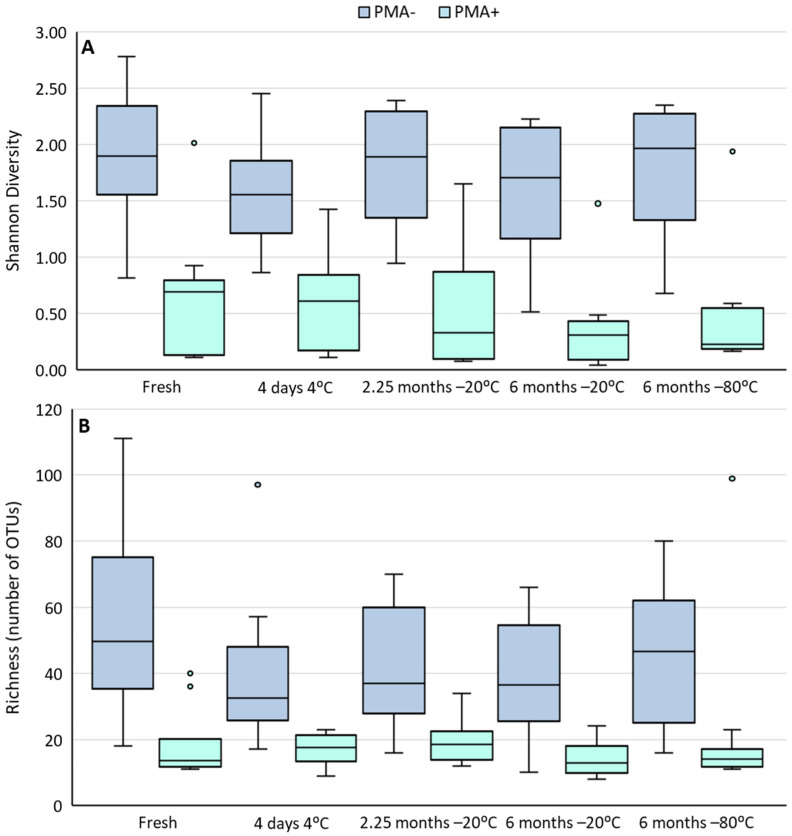
Shannon diversity (**A**) and richness (**B**) of human milk samples under different storage conditions (*n* = 10). Dark blue bars represent total DNA from viable and non-viable cells (non-PMA treated samples). Light blue bars represent DNA from viable cells only (PMA-treated samples). Boxes are interquartile range, whiskers are range, and inner lines are medians.

Cold storage of human milk, regardless of temperature or time, resulted in a significant reduction in the concentration of DNA from viable cells (all *p* < 0.0001 compared to fresh samples). Within non-PMA treated samples (DNA from viable and non-viable cells), cold storage, regardless of temperature or time, did not result in a significant change in the concentration of DNA compared to fresh samples. However, samples stored at −80 °C consistently had almost twice as much DNA as fresh samples (median of fresh: 0.60 ng/µL (IQR 0.55 ng/µL), −80 °C: 1.10 ng/µL (IQR 1.03 ng/µL), unadjusted *p*-value 0.015, adjusted *p*-value 0.19) (Figure 2). These −80 °C stored samples had a significantly higher concentration of DNA compared to those stored under other cold storage conditions (all *p* ≤ 0.003).

### 3.3. Cold Storage Alters the Composition of the Human Milk Microbiome

Cold storage of human milk samples did not alter the alpha diversity (richness or Shannon diversity) of PMA-treated or untreated samples (Figure 2). However, richness was significantly lower in PMA-treated samples compared to non-PMA treated samples under every storage condition (all *p* ≤ 0.0004). Shannon diversity was also lower in PMA-treated samples compared to non-PMA treated samples; however, this trend did not reach statistical significance.

**Figure 3:**
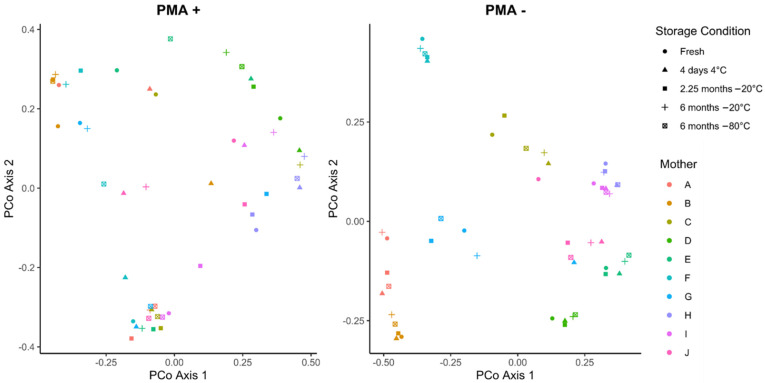
PCoA plot of Bray–Curtis distances of PMA-treated and untreated human milk samples stored under different cold storage conditions (*n* = 10).

Cold storage did not alter the community structure of PMA-treated or untreated human milk (Figure 3). While samples tended to cluster by mother, no clustering was observed by storage condition, regardless of PMA treatment (PERMANOVA all *p* > 0.2). However, the overall bacterial community was significantly different in PMA-treated compared to non-PMA treated fresh and −80 °C stored samples (PERMANOVA *p* = 0.0001 and *p* = 0.0078, respectively).

**Figure 4:**
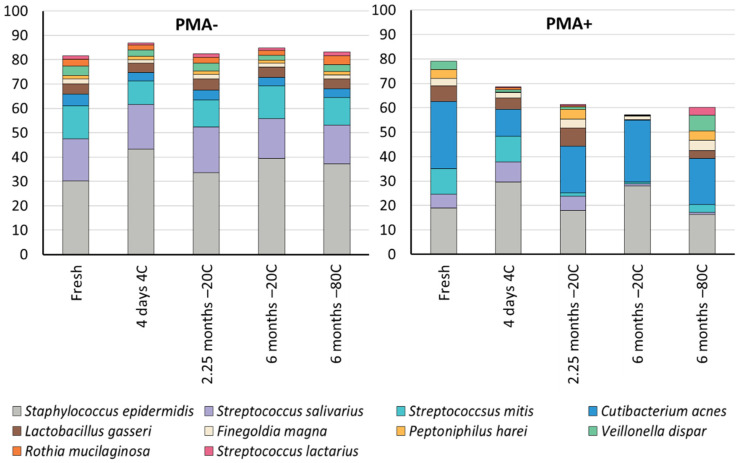
Relative abundance (%) of the ten OTUs which made up ≥1% of the total relative abundance in these samples. Species assignments for each OTU are indicated in the legend.

Ten OTUs made up ≥ 1% total relative abundance within these samples (Table 2). Similar to previous studies, we found that the human milk microbiome was relatively simple, with just three OTUs, mapping to *Staphylococcus* and *Streptococcus* spp., making up an average of 61% of the fresh, untreated samples (Figure 4). Within non-PMA treated samples, cold storage significantly altered the relative abundance of four of the top ten OTUs compared to fresh samples (*Staphylococcus epidermidis*, *Finegoldia magna*, *Peptoniphilus harei*, and *Veillonella dispar*) (all *p* ≤ 0.0306). However, it should be noted that the size of this effect for all OTUs but one (mapping to *Staphylococcus epidermidis*) was small (0.1–1.8% change) (Table 2). Differences between different cold storage conditions were observed for seven of the top ten OTUs (all *p* ≤ 0.0402). However, again, the effect sizes were quite small (0.1–3.6% change). Further studies are required to determine whether this degree of change is biologically impactful.

Within PMA-treated samples, two of the top ten OTUs were absent from the fresh samples. Five of the eight remaining OTUs exhibited significant changes in their relative abundance in fresh compared to cold stored samples (all *p* ≤ 0.0306) (Table 2). Additionally, differences between different cold storage conditions were observed for eight of the top ten OTUs (all *p* ≤ 0.0398) Effect sizes within PMA-treated samples were larger than those seen in non-PMA treated samples (0.2–13.4% change).

## 4. Discussion

Here we demonstrate that cold storage of human milk has no significant impact on the richness, Shannon diversity, nor Bray–Curtis diversity of the viable bacterial community. The fact that viable bacterial richness was not reduced by cold storage suggests that infants fed stored expressed breast milk are exposed to the same number of different viable bacterial taxa as those fed fresh breast milk. However, the composition of the viable microbiome in these samples was significantly altered by cold storage. We observed significant changes in the relative abundance of viable *Streptococcus salivarius*, *Streptococcus mitis*, *Cutibacterium acnes*, *Lactobacillus gasseri*, and *Veillonella dispar* in cold stored samples compared to fresh samples. This finding has implications for storage of expressed breast milk in the home, as such compositional changes may impact infant bacterial colonisation and infant health. Strains of *L. gasseri* have previously been shown to be shared between paired human milk and infant fecal samples, suggesting that it is vertically inherited via breastfeeding [24,25]. Feeding of stored expressed milk may therefore alter this sharing of *L. gasseri* between mothers and their breastfed infants. Similarly, a recent study of paired human milk and infant fecal samples from the CHILD cohort reported that taxa such as Streptococcus and *V. dispar* co-occur in paired human milk and infant fecal samples, and that this co-occurrence is reduced if the infant is fed expressed breast milk [26]. Here, we found that DNA from viable *S. mitis*, the most dominant Streptococcus species in our fresh PMA-treated samples, was significantly reduced after storage at −20 °C for 2.25 or 6 months (by 85.4% and 92.2%, respectively). Similarly, storage at 4 °C for 4 days, −20 °C for 2.25 months, and −20 °C for 6 months, resulted in a significant reduction in the relative abundance of viable *V. dispar* (by 72.7%, 69.7%, and 97%, respectively). Cold storage of expressed breast milk may therefore explain the reduced co-occurrence of these taxa in infants fed expressed breast milk from the CHILD cohort study. Together with the results of Fehr et al., our findings suggest that feeding of frozen or refrigerated milk samples could potentially have an impact on infant gut microbiome colonisation dynamics. Our findings also have implications for the storage of human milk samples in a laboratory setting, as cold storage of such samples may lead to compositional shifts in viable bacterial communities, distorting the results of culture-based or transcription-based microbiome studies. Changes in the composition of the viable microbiome were even observed after storage at −80 °C, with significant decreases in the relative abundances of *S. salivarius*, *S. mitis*, and *C. acnes*, and a significant increase in the relative abundance of *V. dispar* compared to fresh samples. These changes are notable given the widespread reliance on −80 °C storage to preserve microbiome samples.

Our finding that cold storage resulted in a significant reduction in the total quantity of DNA from viable cells suggests that human and/or bacterial cells in human milk are lost during cold storage. Similarly, Zonneveld et al. reported that storage of human milk for 2 h at 37 °C, room temperature, 4 °C, or −80 °C resulted in a significant decline in cell viability [27]. Our results also support the findings of Arahbi et al., which showed a significant reduction in total bacterial cell counts after 1, 3, 6, and 9 months of storage at −20 °C [16]. Collectively, the available data suggests that human and bacterial cell viability is adversely affected by both warm and cold storage. Fresh human milk contains the highest titres of viable cells and DNA, with implications for infant feeding practices and human milk studies.

In addition to investigating the impact of cold storage on the viable microbiome of human milk using PMA, we also investigated the effect on the total bacterial DNA profile. This is important as metataxonomic studies of the human milk microbiome assess bacterial DNA profiles, without differentiation of DNA from viable and non-viable cells. We were therefore interested in assessing the impact of cold storage on the total bacterial DNA profiles of human milk. There was no difference in the richness, Shannon diversity, nor Bray–Curtis diversity of the total bacterial DNA profile. However, numerous small-scale but statistically significant differences were detected in the relative abundance of the ten most abundant taxa after cold storage. These data suggest that minor compositional differences may arise in metataxonomic studies of the human milk microbiome if the samples have undergone cold storage. Human milk samples that are to undergo metataxonomic analysis should therefore be stored in a uniform manner to minimise the effect of different storage conditions on the bacterial DNA composition. These findings also have implications for infants fed frozen or refrigerated expressed breast milk. A large portion of the taxa in fresh milk samples were non-viable (average 67.3% fewer OTUs in PMA-treated fresh samples compared to untreated fresh samples). These non-viable bacteria may play a biological role in the infant gut, by exposing the developing mucosal immune system to a range of different bacteria in a non-threatening manner. Therefore, differences in the composition of the total bacterial DNA profile in cold stored samples may have implications for early-life exposure to living and dead microbes. 

Interestingly, total DNA concentration significantly increased after storage at −80 °C in non-PMA treated samples (Figure 1). Compared to fresh samples, −80 °C stored samples contained approximately twice as much dsDNA. This finding was not reflected by similar increases in the PMA-treated samples, suggesting that an increase in DNA from non-viable cells or cell-free DNA had occurred. This additional DNA may be a result of extracellular vesicle (EV) release as a consequence of cellular stress from freezing at such low temperatures. EVs are lipid-membrane bound compartments released by cells into the surrounding environment. EVs can contain various types of nucleic acid cargo, including dsDNA [28,29,30]. Temperature stress can induce cells to release EVs into their environment [31]. In fact, cold storage of human milk at −80 °C has previously been shown to induce EV formation [27]. This is an important finding, since milk samples are typically stored at −80 °C prior to analysis in lab settings. This may result in inflated estimates of dsDNA quantities due to increased production of EVs. Recently, gram-positive bacteria (the major bacteria in human milk) have been shown to release membrane vesicles with DNA on their surface [32], which may have been detected by our dsDNA quantification here. Importantly, these membrane vesicles were shown to contain immunostimulatory DNA, RNA, and peptidoglycan, that activated innate immune receptors [32]. Such bacterial membrane vesicles may therefore be of importance in early-life immune programming, with differences in fresh and cold stored milk possible.

## 5. Conclusions

In summary, here we demonstrate that cold storage alters the composition, but not the richness or diversity, of the viable and total bacterial DNA profiles of human milk. These findings have significant implications future studies of the human milk microbiome. It is important to note that while we observed changes in the composition of the viable microbiome after cold storage, we did not measure the effects of these on infant colonisation or health. Further, cold storage of human milk following the ABM’s recommendations does not impact other important nutrient or bioactive components of human milk. While this study was limited to a small sample size (*n* = 10 samples, *n* = 43 survey responses), the results nevertheless provided proof of concept and impetus for larger studies on this topic. Given that this was the first study to use a molecular approach to examine the effect of cold storage on the milk microbiome, duplicate processing of the samples may have strengthened the conclusions; however, we do not expect such a duplicate analysis to change our findings. Due to our limited sample size, we are not able to provide recommendations for storage conditions for expressed breast milk. However, it is notable that milk stored at −20 °C for 6 months (the ABM’s recommended maximum storage time) was strongly dominated by just two species, *S. epidermidis* and *C. acnes*. Overall, no cold storage condition tested here preserved the composition of the viable microbiome.

## Figures and Tables

**Table 1:** Cold storage times reported in a survey of 43 mothers who expressed and stored milk for their infants.

	Fridge	Freezer
Average (SD)	1.8 (1.2) days	2.25 (1.74) months
Minimum	5 h	4 days
Maximum	6 days	6 months

**Table 2:** Mean relative abundance (%) of the top ten OTUs in PMA-treated and untreated human milk samples stored under various conditions. Species to which these OTUs map are provided. Statistically significant differences between OTUs at different storage conditions are denoted by superscript letters. Asterisks indicate samples which did not meet the >10% prevalence filter.

Non-PMA Treated Samples	Fresh	4 Days 4 °C	2 Months −20 °C	6 Months −20 °C	6 Months −80 °C
*Staphylococcus epidermidis*	30.2 ^a,b^	43.2 ^a^	33.6	39.5 ^b^	37.2
*Streptococcus salivarius*	17.4	18.3	18.8 ^a^	16.3	16.0 ^a^
*Streptococcus mitis*	13.4	9.8 ^a^	10.9	13.4 ^a^	11.2
*Cutibacterium acnes*	4.8	3.4	4.2	3.5	3.7
*Lactobacillus gasseri*	4.4	3.8 ^a,b,c^	4.6 ^a,d,e^	4.2 ^b,d,f^	4.0 ^c,e,f^
*Finegoldia magna*	2.0 ^a,b^	1.4 ^a,c^	1.9 ^b,c^	1.6 *	1.6
*Peptoniphilus harei*	1.5 ^a,b,c^	1.4 ^a,d,e^	1.3 ^b,d,f^	1.3 *	1.4 ^c,e,f^
*Veillonella dispar*	3.8 ^a^	2.6	3.3 ^b^	2.0 ^a,b^	2.7
*Rothia mucilaginosa*	2.8	2.2 ^a^	2.4 ^b^	1.9 ^c^	3.6 ^a,b,c^
*Streptococcus lactarius*	1.5	0.7	1.4	1.1	1.6
PMA treated samples	Fresh	4 days 4 °C	2 months −20 °C	6 months −20 °C	6 months −80 °C
*Staphylococcus epidermidis*	19.0	29.6 ^a^	17.9	27.9	16.2 ^a^
*Streptococcus salivarius*	5.6 ^a,b,c,d^	8.3 ^a,e,f^	5.8 ^b,g,h^	0.9 ^c,e,g,i^	0.9 ^d,f,h,i^
*Streptococcus mitis*	10.3 ^a,b,c^	10.3 ^d,e,f^	1.5 ^a,d,g,h^	0.8 ^b,e,g^	3.2 ^c,f,h^
*Cutibacterium acnes*	27.6 ^a^	11.1 ^b,c^	19.1	25.2 ^b,d^	19.0 ^a,c,d^
*Lactobacillus gasseri*	6.3 ^a,b^	4.6 *	7.4 ^a,c^	0.3 ^b,c^	3.1 *
*Finegoldia magna*	3.2 *	2.3 *	3.7	1.3 *	4.4
*Peptoniphilus harei*	3.6 *	0.2 *	4.0 ^a^	0.3 *	3.8 ^a^
*Veillonella dispar*	3.3 ^a,b^	0.9 *	1.0 ^a,c^	0.1 *	6.4 ^b,c^
*Rothia mucilaginosa*	0.0 *	0.8 *	0.5	0.0 *	0.0 *
*Streptococcus lactarius*	0.0 *	0.3 *	0.5 ^a^	0.1 *	3.3 ^a^

## Data Availability

FASTQ sequences have been deposited to NCBI SRA (PRJNA758749).
